# Potential Role of Fluoride in the Etiopathogenesis of Alzheimer’s Disease

**DOI:** 10.3390/ijms19123965

**Published:** 2018-12-09

**Authors:** Marta Goschorska, Irena Baranowska-Bosiacka, Izabela Gutowska, Emilia Metryka, Marta Skórka-Majewicz, Dariusz Chlubek

**Affiliations:** 1 Department of Biochemistry and Medical Chemistry, Pomeranian Medical University in Szczecin, Powst. Wlkp. 72, 70-111 Szczecin, Poland; irena.bosiacka@pum.edu.pl (I.B.-B.); emilia_metryka@o2.pl (E.M.); dchlubek@pum.edu.pl (D.C.); 2 Department of Biochemistry and Human Nutrition, Pomeranian Medical University in Szczecin, Broniewskiego 24, 71-460 Szczecin, Poland; izagut@poczta.onet.pl (I.G.); marta_skorka@o2.pl (M.S.-M.)

**Keywords:** Alzheimer’s disease, fluoride, neuroinflammation, reactive oxygen species, cyclooxygenases, antioxidant enzymes, apoptosis

## Abstract

The etiopathogenesis of Alzheimer’s disease has not been fully explained. Now, the disease is widely attributed both to genetic and environmental factors. It is believed that only a small percentage of new AD cases result solely from genetic mutations, with most cases attributed to environmental factors or to the interaction of environmental factors with preexistent genetic determinants. Fluoride is widespread in the environment and it easily crosses the blood–brain barrier. In the brain fluoride affects cellular energy metabolism, synthesis of inflammatory factors, neurotransmitter metabolism, microglial activation, and the expression of proteins involved in neuronal maturation. Finally, and of specific importance to its role in Alzheimer’s disease, studies report fluoride-induced apoptosis and inflammation within the central nervous system. This review attempts to elucidate the potential relationship between the effects of fluoride exposure and the pathogenesis of Alzheimer’s disease. We describe the impact of fluoride-induced oxidative stress and inflammation in the pathogenesis of AD and demonstrate a role for apoptosis in disease progression, as well as a mechanism for its initiation by fluoride. The influence of fluoride on processes of AD initiation and progression is complex and warrants further investigation, especially considering growing environmental fluoride pollution.

## 1. Introduction

Alzheimer’s disease (AD) is a progressive, irreversible neurodegenerative disease and one of the most common causes of dementia. Its clinical symptoms, including the impairment of memory and cognitive functions, are caused by neuronal loss, primarily in the hippocampus and neocortex [1]. Characteristic pathomorphological signs of Alzheimer’s disease include neurofibrillary tangles (NFTs) and amyloid plaques (AβPs), also known as senile plaques. Additionally, pathomorphological studies on AD brains reveal the presence of amyloid neuropathy, granulovacuolar degeneration, synaptic pathology, white matter rarefaction, transactive response DNA-binding protein 43 (TDP-43) pathology, and neuroinflammation [2,3].

Although the etiopathogenesis of AD has not been fully explained, a distinction has been made between its sporadic (sAD) and familial forms (fAD), and the disease is now widely attributed both to genetic and environmental factors [4].

## 2. The Role of Environmental Factors in AD Etiopathogenesis

At present, it is believed that only a small percentage of new AD cases result solely from genetic mutations, with most cases attributed to environmental factors or to the interaction of environmental factors with preexistent genetic determinants [5]. Studies of AD risk factors have been largely inconclusive; however, they have succeeded in generating a list of potential risk factors and demonstrating that the role of these environmental factors in the development of AD is equally important as that of previously established associated genetic mutations [5,6]. Like other neurodegenerative diseases, risk factors include hypertension, hyperhomocysteinemia, hyperlipidemia, and exposure to pesticides and certain metal ions. Additionally, growing attention has highlighted the combination of certain factors. For example, high-fat/high energy diets have been shown to lead to increased aluminum (Al) concentrations in plasma and result in increased concentrations in the brain due to the ability of Al to cross the blood–brain barrier (BBB) [6]. Al has also been shown to alter BBB functions, increasing permeability for nonmetals, such as fluoride [7]. Furthermore, fluoride is known to spontaneously form complexes with trace quantities of aluminum in aqueous environments [8,9]. The roles of Al and F, mainly as AlFx and NaF, have been the subject of extensive investigation in the etiopathogenesis of AD and other human and animal diseases [8,10].

## 3. Neurobiological Processes Leading to AD

Several theories have endeavored to explain the development of AD-related pathomorphological changes in the brain, such as the cholinergic hypothesis (including altered glutamatergic transmission), amyloid aggregation theory, tau protein theory, and oxidative stress theory, of which amyloid β aggregation and tau hyperphosphorylation are currently considered to play the most crucial roles [4].

### 3.1. Cholinergic Hypothesis

Altered cholinergic function, implicated in age-related memory loss since the 1980s [11], is one of the most salient changes observed in AD development [12]. The cholinergic hypothesis was one of the earliest explanations for the etiopathogenesis of AD [4] and serves as the basis for most current AD treatment strategies [13], suggesting that dysfunction of acetylcholine-containing neurons results in the disruption of cognitive processes [13]. This led to the definition of AD as primarily a neurodegenerative process resulting from selective destruction of cholinergic neuronal aggregations within brain structures (including the hippocampus, frontal cortex, amygdala, nucleus basalis, and medial septum, as well as within the areas responsible for memory, learning, and mnemonic processes) [4,13]. This selective depletion of cholinergic neurons in affected brain areas presents as a reduction in cholinergic markers, i.e., acetylcholinesterase and acetyltransferase [14,15]. These changes consequently lead to a reduction in the number and density of nicotinic acetylcholine receptors in AD patients, as well as a reduced expression of subunits α3, α4, and α7 in the cerebral cortex and hippocampus, increased choline uptake, reduced expression of muscarinic acetylcholine receptors, impaired acetylcholine secretion, abnormal axonal transmission, and impaired neurotrophin support [4]. Importantly, in AD patients cholinergic receptors bind amyloid β, disrupting receptor function [16]. Based on brain biopsies of AD patients investigating acetylcholinesterase activity, choline uptake, and acetylcholine synthesis activity, it was confirmed that cholinergic denervation already occurs in the early stages of the disease. Post-mortem studies have confirmed a positive correlation between cholinergic denervation and advancement of memory disorders [17].

### 3.2. Glutamatergic Hypothesis

The glutamatergic hypothesis is an alternative to the cholinergic hypothesis [17], whereby cholinergic dysfunction in AD is explained by abnormalities in related glutamatergic transmission [18].

Glutamate, the anionic form of the amino acid glutamic acid, is the most abundant neurotransmitter in the human brain;—it mediates most excitatory neurotransmission and plays a key role in the processes of memory creation and learning. It also displays neurotoxic properties in animal studies, wherein it leads to the formation of neurodegenerative lesions similar to those observed in the human brain in AD [17,19]. Under normal conditions, glutamatergic transmission in the hippocampus is associated with the generation of a cytosolic calcium ion signal responsible for various synaptic plasticity phenomena including the consolidation of learning and memory processes [4]. However, in pathological situations neurodegeneration occurs as a result of N-methyl-D-aspartate (NMDA) glutamate receptor hyperactivation causing a sustained increase in intracellular calcium, chlorine and sodium ions, thereby leading to excessive depolarization of the postsynaptic membrane [4,18,20]. In AD, alterations in glutamatergic signaling lead to prolonged neuronal exposure to extracellular glutamate, which in turn results in the aforementioned receptor hyperstimulation and excitotoxicity [4].

### 3.3. Oxidative Stress Hypothesis

**Figure 1:**
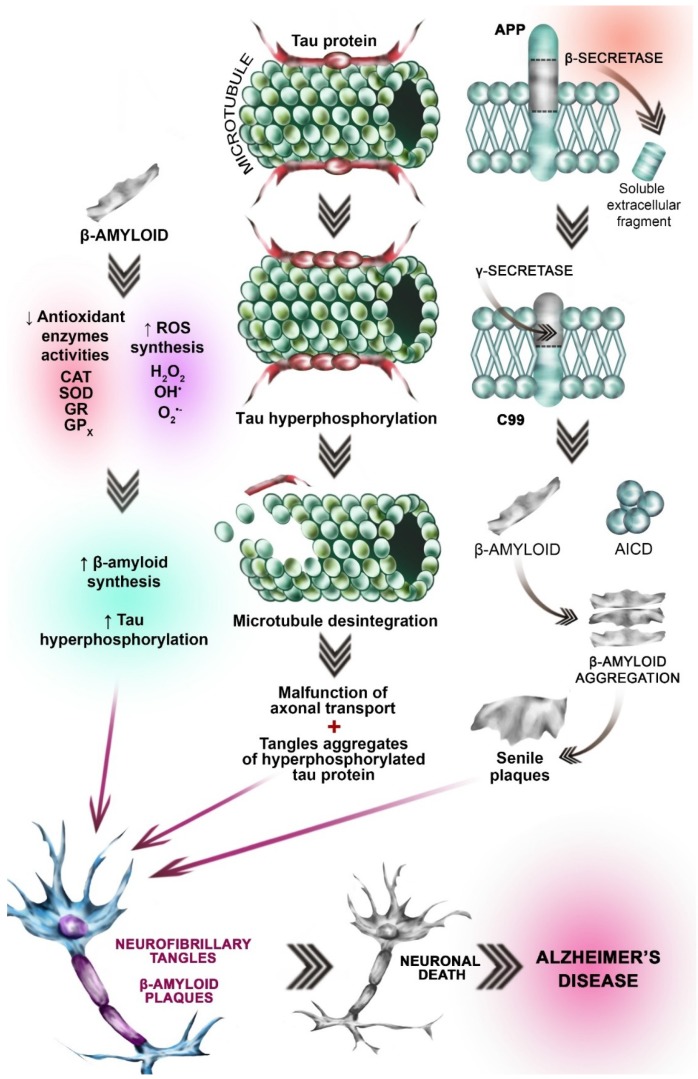
Neurobiological processes leading to AD. Hypothesis concerning OS involvement in AD etiopathogenesis: β-amyloid peptide activates the pathways involved in reactive oxygen species (ROS) synthesis, leading to the increased amount of ROS (left side of the picture). Simultaneously β-amyloid accumulation leads to the antioxidant enzymes’ inhibition (SOD, CAT, GPx, and GR) (left side of the picture. This imbalance between ROS synthesis and antioxidant enzymes activities results in the oxidative stress (OS). Excessive oxidation processes result in tau protein hyperphosphorylation and β-amyloid peptide accumulation (gray arrow, left side of the picture). Tau (τ) protein hyperphosphorylation hypothesis: Under physiological conditions, τ protein is the major neuronal microtubule associated protein. It promotes the assembly of tubulin into stabilizes the microtubules (top picture in the central part of the figure). Under pathological conditions (i.e., OS) Tau becomes hyperphosphorylated. Pathologically-altered τ protein loses its ability to interact with microtubules, leading to disintegration of microtubules (gray arrow, central part of the picture). Hyperphosphorylated Tau is insoluble. Insolubility leads to polymerization into paired helical filaments (PHF), which, together with straight filaments (SF), form neurofibrillary tangles (gray arrow, central part of the picture). Amyloid β aggregation hypothesis: Amyloid precursor protein (APP) is an integral transmembrane protein expressed in many tissues. In AD patients, initial cleavage (by β-secretase) (brown arrow) of the APP results in the extracellular soluble fragment formation. Subsequent cleavage catalyzed by γ-secretase leads to the β-amyloid formation. γ-secretase consists of presenilin, nicastrin, anterior pharynx-defective 1 (APH-1), and presenilin enhancer 2 (PEN-2) β-amyloid, which is insoluble aggregates (right part of the picture, gray arrow) to form In subsequence senile plaques. Another APP derived cleavage product is AICD (the amyloid precursor protein intracellular domain) (green spherical elements). Different AICD levels may contribute to early etiopathological sequences in AD. The processes mentioned above lead to the fibrillary tangles formation, neuronal death and Alzheimer’s disease (three red arrows).

Reactive oxygen species (ROS) are produced in all living organisms as byproducts of normal metabolic reactions and as a result of xenobiotic exposure [21]. Under physiological conditions, they play an important role in cellular signaling throughout the body [22], but in excess they are harmful to all cell types, including nerve cells [21]. Oxidative stress caused by an imbalance between production and elimination of ROS is linked to the pathogenesis of numerous diseases [23,24]. Oxidative stress is not considered to be a phenomenon that itself initiates AD pathogenesis. However, it has been shown to facilitate the progression of the disease and worsen prognosis [24] (Figure 1).

### 3.4. Amyloid β Aggregation Hypothesis

Amyloid β (Aβ) aggregation plays a key role in the etiopathogenesis of AD. The misfolding of Aβ leads to the formation of β pleated sheet-rich aggregates and impairs neuronal function [25].

Aβ is a peptide consisting of 39 to 42 amino acids, some of which form a hydrophobic transmembrane domain. Aβ occurs in several isoforms, of which the most hydrophobic and toxic is 1–42 [24]. It forms extracellular aggregates [26] of varying size dependent on the balance between its synthesis and degradation [27].

Aβ is a peptide product derived from the amyloid precursor protein (APP), a transmembranous protein whose primary function is not known. APP expression increases in cells under increased oxidative stress [4]. Production of Aβ from APP occurs through a sequence of proteolytic cleavages, mediated by secretase enzymes of the disintegrin and metalloproteinase family (ADAM) [4]. In AD patients, initial cleavage leads to the formation of an extracellular soluble fragment (APPsβ) and a longer carboxylic fragment (C99) [4]. This process is mainly catalyzed by β-site-APP-cleaving enzyme (BACE1), whose expression is modulated by oxidative stress, ischemia, trauma, inflammation, and hypoxia—situations common in aging and neurodegenerative disease [4]. γ-secretase (consisting of presenilin, nicastrin, anterior pharynx-defective 1 (APH-1), and presenilin enhancer 2 (PEN-2)) then cleaves the carboxylic fragment at the γ site producing the aggregate-forming Aβ peptide [4] (Figure 1). The amyloid aggregation hypothesis is currently the most widely accepted theory of AD pathogenesis [4].

### 3.5. Tau (τ) Protein Hyperphosphorylation Hypothesis

Studies have shown that the pathological τ protein acts in concert with Aβ in synapse degeneration in AD [28]. Under physiological conditions, τ protein is the major neuronal microtubule associated protein (MAP). Together with MAP1 and MAP2, it is responsible for promoting the assembly of tubulin into microtubules and stabilizing the microtubule network in neuronal axons [29]. In the human brain, τ occurs in six isoforms, differing in their number of binding domains and resulting microtubule stabilizing ability. Under physiological conditions, τ activity has been found to correlate negatively with the degree of its phosphorylation [30]. Further studies revealed that tau activity was affected not only by the degree of phosphorylation but also the phosphorylation site [31]. In the normal human brain, τ is phosphorylated and highly soluble [32]; however, abnormally hyperphosphorylated tau is insoluble [4]. In the brain of AD patients, τ protein is 3 to 4× more phosphorylated than in healthy brains. In addition, τ protein hyperphosphorylation and resulting insolubility leads to polymerization into paired helical filaments (PHF), which, together with straight filaments (SF), form neurofibrillary tangles [29] (Figure 1). Pathologically-altered τ protein loses its ability to interact with microtubules, leading to an increase in free protein and an increase in aggregation and fibrillation, resulting in impairment of axonal function [33].

## 4. Fluoride as a Neurotoxic Agent

Fluoride is widespread in the environment, especially in industrial areas [34]. It easily crosses the blood–brain barrier [35], wherein the accumulation of fluoride disturbs phospholipid metabolism leading to neuronal death [35]. In overexposed women, fluoride can also pass through the blood–placenta barrier to enter the fetal circulation, where it has been shown to inhibit central nervous system development and cause neurodegeneration [36]. In recent years, the mechanism and extent of fluoride’s effect on the nervous system have been the subject of increasing scientific interest [37].

The effect of fluoride exposure on the developing brain (both pre- and neonatal) manifests clinically as memory loss and impairment of cognitive processes. Epidemiological studies showed that children living in areas with excessive fluoride exposure had lower IQ values compared to less exposed children [38]. Industrial workers chronically exposed to fluoride showed a variety of neuropsychiatric symptoms including drowsiness, concentration and learning difficulties, and memory disorders [39,40,41].

Fluoride-induced abnormalities are associated with disturbed metabolism of neurons and glial cells. Fluoride accumulation in the hippocampus has been found to contribute to neuronal degeneration and altered oxygen metabolism, promoting the formation of ROS, and inducing damaging oxidative stress [42,43,44].

**Figure 2:**
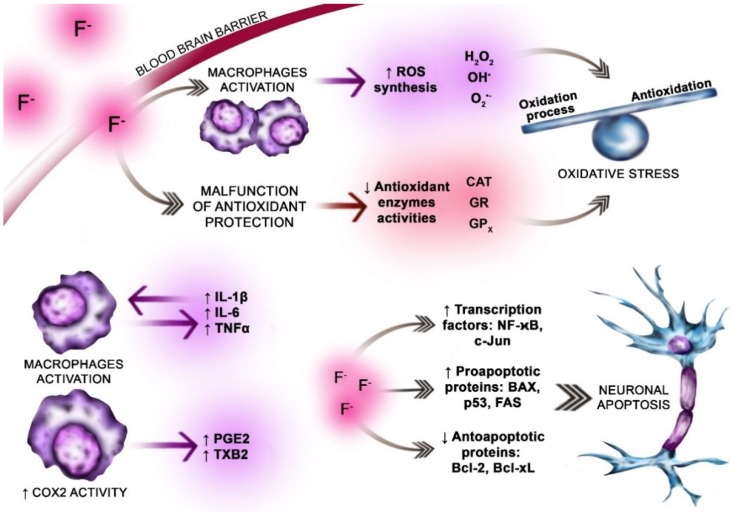
The pro-oxidative and pro-inflammatory effect of fluoride exposure on the brain. Fluoride ions easily cross the blood–brain barrier (dark gray arrow, pink highlight). Within the brain fluoride influences normal metabolism of the neurons and glial cells. The fluoride effect on the nervous system is complex and varied. Fluoride is well documented pro-oxidative factor. Promotes oxidative stress. It enhances reactive oxygen species ROS (H_2_O_2_, OH^−^, and O_2_^•−^) synthesis by activated macrophages (violet highlight). F^−^ weakens the antioxidants’ function by inhibiting the actions of antioxidant enzymes superoxide dismutase (SOD), catalase (CAT), glutathione peroxidase (GPx), and glutathione reductase (GR) (light red highlight). Excessive ROS production and simultaneous impaired antioxidative enzymes’ action leads to the oxidative stress (OS). OS is an imbalance between oxidation and antioxidation processes (blue balance beam). F^−^ enhances the neuroinflammation in the brain. Fluoride dependent stimulation of the pro-inflammatory cytokines synthesis (IL-6, TNF-α, and IFN-γ) is a key step in the inflammation process development. In physiological conditions low concentration of IL-6 in the brain is observed (violet arrows, violet highlight). The increase in the IL-6 is noticed in the inflammation or neurodegenerative diseases. Excessive production of IL-1β and TNF-α is observed in neuroinflammation and neurodegenerative diseases. Overproduction of cytokines in response to fluoride exposition results in inflammation development and neurodegeneration. Fluoride exposition, i.e., due to the inflammatory cytokines increase, leads to the increased activity of the enzymes involved in inflammation (i.e., COX-2) and subsequent production of prostanoids: prostaglandin E2 (PGE2) and thromboxane A2 (TXB2) (violet arrow, violet highlight). F^−^ in the brain increases apoptosis rate by activating the transcription factors (NF-κB, c-JUN) and proapoptotic proteins BAX, FAS, and p53. Simultaneously F^-^ inhibits antiapoptotic proteins synthesis BCL2, BCL-XL (pink highlight, dark gray arrows).

More recent studies have shown that the effect of fluoride on the central nervous system may be extremely varied and complex. In addition to its pro-oxidative effect, fluoride has demonstrated an influence on the activity of antioxidative enzymes, further encouraging damaging levels or ROS [42,45]. Fluoride has also been found to affect cellular energy metabolism, synthesis of inflammatory factors, neurotransmitter metabolism, microglial activation, and the expression of proteins involved in neuronal maturation. Finally, and of specific importance to its role in Alzheimer’s disease, studies report fluoride-induced apoptosis and inflammation within the central nervous system [45] (Figure 2).

## 5. The Role of Fluoride in the Pathogenesis of Alzheimer’s Disease

### 5.1. Oxidative Stress

The brain is predisposed to excessive ROS production and oxidative damage due to its high content of polyunsaturated fatty acids and redox-active metals (Cu and Fe) [23] as well as its high metabolic rate, characterized by high oxygen consumption (20% of basal oxide consumption), and low regenerative capacity [46] compared to other organs.

Elevated oxidative damage in the brain of AD patients is related to the accumulation of amyloid-β (Aβ) and deposition of neurofibrillary tangles and neutrophil threads. Both Aβ and amyloid β precursor protein (APP) have the ability to oxidize copper ions, resulting in the release of hydrogen peroxide [23].

Both the risk of Alzheimer’s disease and levels of oxidative stress increase with age [47]. A study involving rats of increasing ages (7-, 14-, and 21-day-old, adults: 3–6-month-old, and aging: 24-month-old) showed elevated ROS synthesis in the brain of adults and in individual brain areas of aging individuals [48]. This increase in ROS in the brain is linked to disorders of ROS production and/or elimination [49]. Evidence of increased oxidative stress includes increased levels of Cu, Hg, Al, and Fe in brain regions affected by AD-related neurodegeneration and Aβ aggregation, increased lipid peroxidation and reduced polyunsaturated fatty acids, increased protein and DNA oxidation, decreased energy metabolism, and advanced glycation end-products in neurofibrillary tangles [23,50,51,52].

Markesbery and Lovell found that four-hydroxy-nonenal (HNE), a product of lipid peroxidation, is a neurotoxin whose concentration is elevated in the ventricular fluid of AD patients. They also reported an increase in HNE levels in the brain regions most affected by degeneration in AD [51], indicating a potential role in pathogenesis.

Further evidence of oxidative stress comes from the observation by Ansari et al. of an increased concentration of carbonylated proteins in parts of the brain with pathomorphological changes resulting from AD (i.e., the frontal lobe, parietal lobe, and hippocampus) [52].

Living organisms are protected against the effects of excessive ROS by antioxidants, including antioxidant enzymes and glutathione (GSH). Antioxidant enzymes are divided into those directly involved in the inactivation of ROS—i.e., catalase (Cat), superoxide dismutase (SOD), and glutathione peroxidase (GPx)—and glutathione reductase (GR), which catalyzes the reduction of oxidized glutathione. Reduced glutathione is an electron donor in the peroxidation reaction catalyzed by GPx. GSH also has the ability to directly interact with ROS [53,54,55]. While alterations in ROS synthesis and oxidative stress in AD have been confirmed in both animal and human models, data on changes in the activity of antioxidant enzymes are inconclusive [48,49,51,56]. 

Though specific findings are inconsistent, the activity and levels of antioxidant enzymes are altered in AD. A review by Niedzielska et al. describes an increase in SOD activity in the hippocampus and amygdala of AD patients, but decreased levels of SOD, GPX, and Cat in the frontal and temporal cortex and a decreased GSH content in brain and erythrocytes [56,57,58,59]. In addition, lymphocytes of AD patients were characterized by a higher level of Cu/ZnSOD mRNA [60] compared to both healthy subjects and those with Parkinson’s disease.

It is generally recognized that substances that support ROS scavenging or increase the activity of antioxidant enzymes have a positive effect in the treatment of Alzheimer’s disease symptoms. A proposed strategy for the treatment of neurodegenerative diseases associated with abnormal oxidation involves the use of medicinal substances with antioxidant enzyme activity [61].

### 5.2. The Role of Fluoride

Fluoride has a well-established prooxidant effect in cells [62,63,64]. Shuhua et al., in studies with murine microglial BV-2 cells, showed that the toxic effects of fluoride on the nervous system can at least partly be attributed to microglial activation, leading to increased synthesis of ROS and RNS (reactive nitrogen species) and resulting in oxidative stress [65]. This is confirmed in a study by Saralakumari et al. showing a relationship between chronic exposure to fluoride and increased oxidative stress in humans [66].

The pathomechanism of fluoride toxicity also involves its effect on several enzymes, including antioxidant enzymes. The exact nature of this influence is not clear, but fluoride exerts a predominantly inhibitory effect on antioxidant enzyme activity [42]. A study conducted by Zhang et al. endeavored to explain the mechanism of fluoride neurotoxicity. Rat hippocampal neurons cultured in the presence of sodium fluoride (20, 40, and 80 mg/L) for 24 h, showed a significant decrease in GPx activity and decreased GSH concentration. A decrease in SOD activity was also observed in cells incubated with high concentration of NaF [44]. Increased Cat activity [67] was observed in brain tissues of young rats given fluoridated drinking water, which can be explained as the activation of protective mechanisms against the effects of excessive oxidation [67,68]. A study conducted by Pal and Sarkar on rats exposed to fluoride at 20 mg/kg showed a reduction in GSH concentration and a decrease in the activity of Cat, SOD, and GPx in brain tissues [68].

In vitro studies on macrophages obtained from THP-1 cell line monocytes (not yet published by our team) showed that fluoride not only exerted a negative effect on the activity of antioxidant enzymes, but also negated the positive effect of acetylcholinesterase inhibitors on these enzymes (Cat, SOD, GPx, and GR) and lowered GSH concentration [69]. This effect was observed after application of fluoride at a low (3 µM) concentration reflecting chronic environmental exposure levels [69].

## 6. Inflammation in AD

Alzheimer’s disease is one of many diseases associated with inflammation [69]. The factors and pathways involved in neurodegeneration are not well understood; however, recent data suggest that inflammation is central to this process [70].

The involvement of inflammation in the pathogenesis of neurodegenerative diseases, (including AD) has been indirectly confirmed by the results of epidemiological studies demonstrating prevention or inhibition of the progress of AD in patients after use of anti-inflammatory drugs [71]. In their 2004 review, Szekely et al. cited data showing reduced AD incidence in persons taking anti-inflammatory drugs for other ailments [72].

It is believed that inflammation in itself is not the primary initiator of neurodegeneration in AD and other neurodegenerative diseases. However, the long-term upregulation of inflammatory response resulting from activation of microglia and astrocytes in neurodegenerative disease suggests an important role for neuroinflammation in neuronal dysfunction and death [70]. Current literature extensively describes the inflammatory mechanisms which are active in the development and progress of AD [73].

### 6.1. Fluoride vs. Nuclear Factor κB (NF-κB)

The mechanisms underlying neuroinflammation are very complex and difficult to identify. However, it is known, for example, that inflammation in the central nervous system induces the activation of nuclear transcription factor κB (NF-κB), a major transcription factor regulating the expression of genes responsible for immune response [74]. Literature also suggests an important role for NF-κB in inflammation resulting from the interaction between microglia and astrocytes [75,76]. Fluoride has been shown to stimulate in vitro NF-κB activity in BV2 microglia [34]. Additionally, NF-κB can be activated by IL-1 interleukin and tumor necrosis factor α (TNF-α), both of which are produced in the brain in response to exposure to fluoride [77,78]. Activation of NF-κB can have numerous results, including stimulation of nitric oxide synthase activity, increased NO production, and induction of expression of cyclooxygenase 2 (COX-2), an enzyme that plays a key role in the induction and progression of neuroinflammation [77].

### 6.2. Fluoride vs. Proinflammatory Cytokines

Fluoride has been linked to many aspects of the neuroinflammatory process, including stimulation of cytokine secretion and direct influence on macrophages and microglia, whose activated forms in turn constitute an important source of proinflammatory factors in AD [79].

Since the 1990s, increased expression of inflammatory markers—including acute phase proteins such as α1-antichymotrypsin and proinflammatory cytokines such as interleukin-1 (IL-1), interleukin-6 (IL-6), and TNF-α—has been implicated in AD pathogenesis [80,81,82,83]. This was confirmed in a study on Alzheimer amyloid precursor protein (APP)-transgenic mice (APP-Tg), where, following injection of LPS (lipopolysaccharide) into peripheral circulation, AD mice showed a significantly higher concentration of IL-6 than wild-type mice [84]. It has also been established that overproduction of IL-1, IL-6, and TNF-α stimulates the synthesis of Aβ [85] and IL-1 is associated with both the initiation and spread of neuroinflammation in AD [86].

An in vitro study by Wang et al. on HeLa cells showed a fluoride-induced stimulation of the synthesis of proinflammatory cytokines IL-1β, IL-2, IL-6, and TNF-α [87]. Activation of microglia in the hippocampus and cerebral cortex was also demonstrated in the rat model where fluoride, by influencing the production of these cytokines, contributed to the formation and progression of inflammation in the brain [88].

### 6.3. Fluoride vs. Neuroinflammation Enzymes

Post-mortem studies on the brains of Alzheimer’s disease patients show that senile plaques are infiltrated by activated microglia cells, which then serve as an important source of cytokines [89,90]. Cytokines released by microglia, by binding to receptors on astrocytes coupled to Ca^2+^-dependent enzymes, may cause the activation of these enzymes, including cytosolic phospholipase A2 (cPLA2) and secretory sPLA2 [91]. Phospholipases A2 catalyze the hydrolysis of the ester bond in the sn-2 position of glycerophospholipids, releasing fatty acids, including arachidonic acid, which further encourages the inflammatory process [92]. In turn, activation of PLA2, and the resulting increased availability of free arachidonic acid, promotes the synthesis of proinflammatory eicosanoids in macrophage cells [93].

The stimulating effect of aluminum fluoride on the activity of phospholipase A2 in macrophages was recognized as early as the 1980s [94]. Gutowska et al. were one of the first to show the stimulating effect of low concentrations of fluoride on PLA2 exocrine activity and subsequent eicosanoid production, specifically prostaglandin E2 (PGE2) and thromboxane A2 (TXA2), indicating increased activity of cyclooxygenases [93]. Subsequently, Chalbot et al. demonstrated that sPLA2 activity in cerebrospinal fluid collected from individuals with diagnosed AD is higher than in cerebrospinal fluid from healthy persons [95]. Brains of AD patients also show increased levels of functional cPLA2 protein and mRNA expression [96,97,98].

Furthermore, ROS—the production of which is increased in AD—activate mitogen-activated protein kinases (MAPK), which in turn activates PLA2. cPLA2 activity is in turn associated with neuronal excitotoxicity, impairment of mitochondrial function, and neuronal apoptosis. Parallel activation of the previously mentioned NF-κB [97]—which is also activated by fluoride in the central nervous system [99]—can further induce expression of sPLA2 and cyclooxygenase 2 (COX-2), and thereby increase inflammation [97,98,99].

Also among the enzymes involved in the development of inflammation in AD, COX-2, also known as prostaglandin-endoperoxide synthase 2, is an important enzyme in the metabolic cascade of arachidonic acid and PLA2 [100]. Increased COX-2 activity has been previously described in the frontal cortex of AD patients [100]. COX-2 is an inducible enzyme whose expression is associated with increased inflammation and various pathological processes [100]. COX-2 is induced by numerous proinflammatory stimuli including inflammatory cytokines and monocyte cells themselves [101]. COX-2 catalyzes the synthesis of prostanoids (PGE2, TXA2) from arachidonic acid and serves as the main source of these molecules during inflammation [102]. These prostanoids then mediate inflammatory upregulation, an important factor in the pathogenesis of AD [69,100].

An important discovery in this field was the identification of a constitutively expressed form of COX-2 within a specific neuronal population, where it supports synaptic activity and long-term plasticity [103]. As a result, some researchers suggest that in order to accurately describe the expression of COX-2 in the nervous system, the term “constitutive expression” should be replaced by “dynamic regulation”, as constant COX-2 expression is observed during normal synaptic activity, while expression increases during convulsions or ischemia [104].

A potential link between COX-2 activity and Alzheimer’s disease pathogenesis was suggested in the 1990s and 2000s [105,106]. It was found that administration of nonsteroidal anti-inflammatory drugs (NSAIDs)—which inhibit COX activity—to AD patients resulted in inhibited progression of clinical symptoms [107]. The study also confirmed an increased expression of the COX-2 gene in the frontal cortex of AD patients compared to healthy subjects [107]. Moreover, synthetic β-amyloid peptides induced COX-2 expression in SH-SY5Y neuroblastoma cells in vitro, suggesting a mechanism for COX-2 upregulation in AD [108]. At the same time, COX-2 has been implicated in processes leading to the formation and progression of both neuritic plaques (NP) and neurofibrillary tangles (NT) [109]. However, conflicting results exist with some evidence suggesting that the number of COX-2 positive neurons decreases with increased severity of AD measured by clinical dementia rating (CDR) [110].

Based on available data, it is likely that COX-2-dependent neurodegenerative effects result from the action of prostanoids produced in the COX-catalyzed reaction [111,112]. The stimulating effect of fluoride on prostanoids production was observed by Schulze-Specking et al., who reported that fluoride promoted the release of arachidonic acid from cell membranes and the synthesis of prostaglandins in rat liver macrophages. Furthermore, fluoride initiated the translocation of protein kinase C from the cytoplasm to cell membranes, indicating that Ca^2+^-dependent protein kinase C is involved in the proinflammatory action of fluoride [113]. The stimulating effect of fluoride on PGE2 production in hepatic macrophages was confirmed by Dieter et Fitzke [114]. A more recent study with human THP-1 macrophages demonstrated that exposure to low fluoride concentrations—which may be considered to reflect “environmental” exposure—led to an increase in PGE2 and TXB2 production [93].

Our team conducted a study on the influence of acetylcholinesterase inhibitors on cyclooxygenase activity in regards to the proinflammatory action of sodium fluoride on macrophages. Many reports indicate that these drugs, which are commonly used in the treatment of AD, may have other mechanisms of action beside inhibition of acetylcholinesterase. Our study confirmed the inhibitory effects of two popular AD drugs, donepezil and rivastigmine, on the production of PGE2 and TXB2 in macrophages, as well as on the expression of COX-1 and COX-2 mRNA and protein. We also demonstrated that the proinflammatory effect of fluoride may be reduced by the combined use of both drugs at their highest concentrations used in our study [69].

### 6.4. Fluoride vs. Neuroapoptosis

Apoptosis has been an important topic in AD research since the 1990s, when a link was suggested between apoptosis and nerve cell loss in AD brains. Deeper understanding of this topic was considered necessary for the development of new therapies [115].

Apoptosis plays a key role in the maintenance and progression of physiological processes (e.g., tissue homeostasis, aging, healing, and embryogenesis) [115,116]. Under physiological conditions, apoptosis pathways are responsible for protecting the body against damage caused by the presence of abnormal or mutant cells. However, disruptions or alterations in normal apoptotic pathways may lead to abnormal or unregulated growth of cells, resulting in pathology and oncogenesis [117]. Excessive apoptosis has long been thought to play a role in the pathogenesis of neurodegenerative diseases, such as Alzheimer’s and Parkinson’s disease [115,118], diseases in which environmental factors such as fluoride seem to be of key importance.

Activation of apoptosis may occur as a result of detection of extensive DNA damage by the DNA repair mechanism. Specifically, this type of DNA damage may be caused by increased oxidative stress such as occurs in AD [115,117,118,119]. Further, oxidative stress causes autocatalytic production of hydroxyl radicals which can induce activation of NF-κB [120,121], a transcription factor with a key regulatory role in apoptosis [122]. Depending on contextual factors (e.g., apoptotic stimulus and cell type), NF-κB can either protect cells against apoptosis or initiate this process [79,121,122]. An in vitro study on rat hippocampal neurons showed increased expression of NF-κB and an increased percentage of apoptotic cells following treatment with sodium fluoride [99]. An influence of fluoride on apoptosis was also demonstrated in in vitro studies carried out on SH-SY5Y neuroblastoma cells, where fluoride induced an increase in caspase-3 concentration and an increase in the expression of Fas, Fas-L, caspase-3, and caspase-8, suggesting that fluoride-dependent damage to neural cells results from—among other reasons—mitochondrial apoptosis due to the Fas-dependent activation of caspase-8 and subsequent activation of caspase-3 [123]. Similarly, the potential proapoptotic effects of fluoride were demonstrated in vivo by Liu et al. where rats exposed to fluoride showed an increased number of apoptotic cells in their brains. Moreover, an increase in phosphorylation of Jun N-terminal kinases (JNK) was observed, suggesting that in this case, the proapoptotic effect of fluoride is mediated by activation of JNK kinases [124]. which in turn trigger the activation of caspases [124,125].

Another study, also conducted on rats, described an increased expression of proapoptotic Bax protein and decreased expression of antiapoptotic Bcl-2 protein in response to fluoride. Analysis carried out by means of the terminal deoxynucleotidyl transferase dUTP nick end labeling (TUNEL) method confirmed an increase in apoptotic processes in brain structures [88].

TUNEL was performed during autopsy on the brains of people with Alzheimer’s disease. Several such studies confirmed DNA fragmentation and in some cases, TUNEL-positive cells showed apoptotic morphology [126,127]. Studies have also found increased expression of antiapoptotic Bcl-2 and Bcl-xl, as well as proapoptotic Bak and Bad, in the temporal cortex of AD patients compared to healthy controls. Extended studies on individual protein fractions have concluded that Bak and Bad [128] are more involved in AD-related apoptosis than Bax. It has been suggested that the balance between proapoptotic (Bax, Bad, and Bak) and antiapoptotic (Bcl-2 and Bcl-xl) proteins may be a key factor for the survival of individual neurons [129]. Also of note in AD-related apoptosis is the role of caspases. Masliah et al. observed increased immunoreactivity of neuronal caspase-3 and Bcl-2 in AD brains [130]. Moreover, neurons displaying DNA fragmentation showed more intense caspase-3 immunoreactivity compared to intact neurons, suggesting apoptotic activity [130].

Transcription factors c-Jun and NF-κB have also been linked to the initiation of apoptosis in AD and the mechanism of proapoptotic action of fluoride. JNK-phosphorylated c-Jun is thought to be involved in neuronal apoptosis, as evidenced by the observed increase in c-Jun and NF-κB expression in AD brains [131,132].

### 6.5. Fluoride Vs. Mitophagy

Mitophagy is a way of controlled elimination of dysfunctional mitochondria by autophagy. This process is highly selective form of autophagy and allows to maintain the proper functioning and networking of mitochondria. Mitophagy is preceded by the fragmentation of mitochondria system. It allows the elimination of the impaired organelle to occur with no influence on the mitochondria network [133]. Mitophagy is suggested to be one of the earliest process during the onset of Alzheimer’s disease [133]. In the AD brains mitochondria may exhibit various abnormalities i.e., morphology pathologies, impaired functioning, increased mutations within the mtDNA or improper activities of the mitochondrial enzymes [134]. APP and Aβ aggregation are suggested to aggravate the pathologies of the mitochondria, i.e., by accelerating the oxidative stress. [134,135,136]. Moreover extensive ROS synthesis enhances Aβ accumulation and subsequent Aβ mitochondrial toxicity itself [56].

Among mechanisms determining the negative fluoride effect on mitochondrion functioning the influencing glucose metabolism in mitochondrion and enhancing oxidative stress are mentioned [45]. Fluoride is involved in ROS production in mitochondria. Excessive ROS synthesis together with impaired functioning of antioxidant enzymes may disrupt the mitochondrion metabolism [45]. Fluoride-induced oxidative stress influences enzymes essential in ATP synthesis process, thus decreasing ATP bioavailability. As the consequence the changes in mtDNA are observed and the cell death occurs. ATP obtaining in mitochondrion can also be altered in consequence to fluoride exposition due to the impaired glucose metabolism in neurons [137,138].

Fluoride is an element with a hypothetical role in the AD etiopathogenesis AD [45]. Concerning the information mentioned above, the toxic effects of fluoride on mitochondria, including hypothetical involvement in mitophagy occurrence, should be taken under consideration during the AD etiopathogenesis [45,137,138].

### 6.6. Potential Roles of Alterations in Zinc and Magnesium Concentrations in Relation to Fluoride-Induced Neurodegeneration

Zinc (Zn) is an essential microelement with a complex role in the organism. Zinc is needed in a process of activation of different proteins (enzymes and receptors) and constitutes structural element in particular proteins [139]. Zn is needful for the proper functioning of signal transduction pathways, which are involved in the gene transcription modulation. Positive effects of Zn action are due to several mechanisms, including the inhibition of ROS synthesis. In the brain zinc is involved in signal transduction pathways, acting as a neurotransmitter [140]. Senescence is a physiological, complex process, which predisposes to Zn deficiencies. AD etiopathogenesis is linked to both increased and decreased zinc content within the organism [141,142].

Excessive accumulation of zinc in the brain induces Tau protein hyperphosphorylation, which in turn affects NMDA receptors (NMDARs) via positive feedback, leading to the excitotoxic death of neurons and production of neurofibrillary tangles. Moreover, excess zinc accumulation is associated with the inhibited ferroxidase activity of APP, which results in the accumulation of bivalent iron in the brain and subsequent intensification of pro-oxidative processes [143]. Another effect consists in the synaptic accumulation of amyloid β oligomers around NMDAR receptors (namely, NR2B units) [141].

Although an increased concentration of zinc in the body is increasingly often mentioned in the aspect of AD etiopathogenesis, zinc deficiency may also contribute to the onset of AD. Reduced zinc concentration in peripheral blood is a factor reducing appetite, which further increases the risk of zinc deficiency in older adults. Also highly significant is the intensification of the inflammation process observed in zinc deficiency [141,144]. Finally, the loss of zinc predisposes to synaptic dysfunction [145] and indirectly activates NADPH oxidase and nitric oxide synthase, resulting in the destruction of mitochondria [146].

Due to the high incidence of Zinc deficiency around the world, its role in fluoride etiopathogenesis of AD should not be neglected [141,145]. Exposure to fluoride is a very likely factor potentiating biochemical changes caused by inadequate levels of zinc. In zinc deficiency, exposure to pro-inflammatory and pro-oxidative fluoride is likely to accelerate and intensify the production of ROS, thus leading to oxidative stress, which may exacerbate the signaling pathways associated with inflammation. Simultaneous exposure to fluoride and zinc deficiency may increase the risk of impaired mitochondrial metabolism and mitophagy. On the other hand, exposure to fluoride together with excessive zinc concentration in the central nervous system could also hypothetically promote AD. Both exposure to fluoride and excess zinc trigger the aforementioned mechanisms, resulting in hyperphosphorylation of the Tau protein, which in turn leads to the neurofibrillary tangles generation (as above) [45,143].

Neurodegenerative diseases onset is in connection with magnesium (Mg) deficiency nowadays [147]. Taking into consideration mechanisms leading to the AD onset it is highly possible that Mg deficiency can determine the initiation and severity of AD after fluoride exposure. Fluoride induced oxidative stress and inflammation as well as Mg deficiency are demonstrated to have a stimulatory effect on BACE1 activity. This enzyme, as mentioned below (part 3.0), is a key enzyme involved in APP transformation into amyloid β [147,148]. Moreover there is evidence recognizing the Mg deficiency as the factor contributing to the initiation of neurogenic inflammation [147,149]. Mg is involved in the modulation of the NMDA receptors functioning. It was also suggested that a reduced Mg level may potentiate the glutaminergic transmission, resulting in intensified excitotoxicity and potentiated inflammatory-related signal cascades [150]. 

Currently no studies directly linking the AD with the exposure to fluoride in combination with zinc alternations (increased or decreased concentration) or Mg deficiency can be found. However, according to a considerable amount of reported data fluoride should be, with high possibility, taken under consideration as the factor intensifying the biochemical changes caused by Zn or Mg homeostasis imbalance. It requires further research.

### 6.7. Potential Roles of Xenometals and Xenobiotics in Relation to Fluoride-Induced Neurodegeneration

Among the others xenometals and xenobiotics of the environmental origins with the supposed role in AD etiopathogenesis, the heavy metals (i.e., lead, Pb), pesticides (i.e., organophosphates and carbamates) are mentioned [151,152].

Heavy metals are suggested to be involved in the pathogenesis of AD. Some of the environmental studies indicated aluminum (Al) to increase the risk of AD occurrence [153]. A meta-analysis of studies published up to 2015 showed that patients with the long-lasting exposure to the Al exerted higher risk of AD development in comparison to the healthy individuals [154]. Although a metanalysis conducted by Virk et Eslick indicated no connection between occupational exposition to Al and occurrence of AD [155]. The metanalysis (up to March 2015) used three studies based on case–control (with a sample size of 1056 patients) [156]. The data concerning the Al presence in the cores of the amyloid or senile plaques have been inconsistent, with some indicating the presence of this element within AD plaques. But the fluorescent examination of the AD hippocampus revealed the Al within the neurons [157].

Excessive collection of Al in the brain results in the increased processes of ROS synthesis together with weakened antioxidant protection and enhanced cellular cascades related to the inflammation are supposed to be involved in AD etiopathogenesis [152]. Aluminum was reported to inhibit in animal in vivo model activities of enzymatic oxidants (CAT, GPx, GR, and SOD) as well as Na^+^/K^+^ ATPase, Mg^2+^ ATPase, and Ca^2+^ ATPase in all brain regions in comparison to healthy control. Aluminum administration decreased the reduced form of glutathione concentration [158]. Morris et al. also indicate that Al exposure could result in apoptosis of a hippocampal and cortical regions. Moreover in their review authors indicate that “physiological/environmental” concentrations of the Al could promote the aggregation of Aβ and the subsequent fibrillary structures deposition [157].

Inflammation enhancement, stimulation of ROS synthesis with simultaneous impairment of antioxidant bioavailability (resulting in oxidative stress), and apoptosis modulation are the common effects for fluoride and Al exposition. Moreover in water environment a formation of Al and fluoride complexes occurs [8]. Exposure to the complexes of Al and may cause more severe disruptions of neurons metabolism than aluminum alone [8]. On the other hand fluoride in a complexed form with Al may exert the properties that are not noticed outside the complexes (i.e., protein G activation) [8].

An element with well-documented neurotoxicity is lead (Pb). The strength of lead’s toxic properties resulted in placing this element among the most hazardous factors [159]. Lead plays a key role during all phases of inflammation, it modulates proinflammatory cytokines (i.e., IL-8), the expression of inflammation enzymes (i.e., COX-2, caspase-1, and NOS2), or affects the synthesis of purines receptors (PX24 and PX27) [159,160,161]. This Pb-induced disruption of immune system functioning is caused by the disturbances on both cellular and hormonal levels [159]. Toxic effects (with the special impact on neurotoxicity) of Pb have been extensively studied in vivo by Gąssowska and Bosiacka et al. The researchers reported that pre- and neonatal exposure to low concentrations of Pb may result in destroying of synapses structure as well as in the changes of key synaptic proteins expression (synaptotagin-1, Syntaxin-1, and SNAP-25 in certain brain parts) [162]. Researchers also showed the relation between perinatal exposure to Pb at low (below threshold level) concentration and alteration in sphingosine-1 phosphate and its receptor expression (S1PR1) levels in various brain regions in rats [163]. Lead was also proven to reduce the number of hippocampal neurons in rats exposed perinatally to low concentrations of Pb [164].

Pb-dependent impairment of brain metabolism with subsequent clinical implications is well described in children, but there are few studies in aging population of humans [165]. Although many papers examining the potential role of Pb in AD etiopathogenesis appeared, there is still a great need to study the potential link between AD and Pb exposure [165]. In humans the Pb concentrations in the plasma were observed to be positively correlated to the worsening of the verbal memory [166]. The data linking Pb exposure with AD mainly concern the animal model. The in vivo studies in rat model revealed that Pb exposure leads to the increased amount and to hyperphosphorylation of Tau protein [167]. Enhanced tauopathy in animal models has also been reported by other groups [168,169]. Pb has been also proven to increase amyloid β and senile plaques formation in transgenic mice and in monkey model [170,171].

The mechanisms linking the Pb neurotoxic effects with the Alzheimer disease show certain correlation with the mechanisms affected by fluoride. Unfortunately there were no studies on cumulative or additive effects of those two toxic agents in nervous system. Gutowska et al. conducted study in an in vitro model (Hep-G2 cells) examining the simultaneous effects of Pb and fluoride on apoptosis, cells vitality, and cells proliferation [172]. The hepatocytes were exposed to the NaF, Pb acetate, or a mixture of both substances. Exposure to the separately used substances resulted in enhanced apoptosis. The apoptosis increase was more excessive after exposure to both agents. Pb extensively decreased cell proliferation index. In a case of simultaneous use of Pb and NaF the abolishing properties on Pb effect were observed [172]

Among xenobiotics involved in the neurodegeneration thus AD etiopathogenesis pesticides are with the particular importance. Pesticides are a heterogenic group of chemical substances used for plant protection against insects or fungi [173]. The related mechanisms of pesticides have been reported to alter tau protein properties, i.e., by stimulating the kinase p-GSK3β (which phosphorylates Tau protein). Moreover mechanism of pesticides neurotoxicity is related to the neurons death in certain brain regions (hippocampus and cortex) leading to the dementia symptoms [151]. As the mechanisms related to the organophosphate-induced neurodegeneration leading to AD also apoptosis induction, impairment of cholinergic system functioning and promoting the β amyloid synthesis and oligomers aggregation are reported [174].

Mechanisms determining pesticide neurotoxicity seem to be related to the same processes or alterations in cell metabolism as in the case of fluoride toxicity. The potential additive effect of pesticides and fluoride exposition should be taken under consideration. Unfortunately no studies describing simultaneous exposure to fluoride and pesticides have been reported. The further investigation concerning the co-influence of pesticides and fluoride on neurotoxicity and neurodegeneration should be conducted. 

This review aimed to show the potential role of fluoride exposure in the etiopathogenesis of AD which is an example of neurodegenerative disease. The data above clearly indicate that despite the influence of fluoride alone, the co-influence of other substances also needs to be examined and searched. The association between the influence of many toxic agents on neurodegenerative disease is a huge problem in the field of public health.

## 7. Conclusions

Alzheimer’s disease is one of the most common causes of dementia. Its clinical presentation, including memory impairment, cognitive disorders, and neuropsychiatric symptoms, results from pathomorphological and pathophysiological changes in the central nervous system.

The research presented in this review clearly indicates that fluoride may play a key role in the induction and development of inflammation in AD and participate in processes of neurodegeneration. Fluoride may promote the synthesis of proinflammatory factors (e.g., prostaglandins and proinflammatory cytokines including IL6, TNF-α, IL1B, IL-4, and IFN-γ), transcription factors (c-Jun and NF-κB), and proapoptotic proteins (Bax, p53, and FAS receptor protein), as well as reduce synthesis of antiapoptotic proteins (BCl-2 and BCLXL).

Moreover, fluoride has been shown to affect the expression and activity of enzymes involved in inflammation (e.g., COX-2) and alter oxidative balance (i.e., modify ROS levels, cause dysfunction in activity and expression of SOD, CAT, GPx, GR, and GSH).

In summary, the influence of fluoride on processes of AD initiation and progression is complex, not yet fully understood, and warrants further investigation, especially considering growing environmental fluoride pollution.

## 8. Perspectives for Further Research

Alzheimer’s disease, characterized by memory impairment, cognitive disorders, and other neuropsychiatric symptoms, is one of the leading causes of dementia and results from pathomorphological and pathophysiological changes within the central nervous system [3]. Long-term research on the pathogenesis of AD has suggested various causes and mechanisms leading to the observed neurodegeneration, with a great deal of attention initially paid to so-called genetic factors [175]. As AD research has evolved, increasing significance has been attributed to the influence of environmental factors exhibiting pro-oxidative, proinflammatory, or proapoptotic effects and demonstrating an impact on neuroinflammation or neurodegeneration. Among these factors, fluoride, an element with multidirectional effects on cellular redox status, has attracted notable attention. Fluoride has been found to increase production of superoxide species [61,63,69]. It also leads to an increase in the activity of enzymes associated with inflammation (sPLA2, COX), increased expression of NF-κB and other proapoptotic factors, and increased activity of JNK.

This review attempts to elucidate the potential relationship between the effects of fluoride exposure and the pathogenesis of Alzheimer’s disease. We describe the impact of fluoride-induced oxidative stress and inflammation in the pathogenesis of AD and demonstrate a role for apoptosis in disease progression, as well as a mechanism for its initiation by fluoride.
